# Noncanonical Sequences Involving NHERF1 Interaction with NPT2A Govern Hormone-Regulated Phosphate Transport: Binding Outside the Box

**DOI:** 10.3390/ijms22031087

**Published:** 2021-01-22

**Authors:** Tatyana Mamonova, Peter A. Friedman

**Affiliations:** Laboratory for GPCR Biology, Department of Pharmacology and Chemical Biology, University of Pittsburgh School of Medicine, Pittsburgh, PA 15261, USA; paf10@pitt.edu

**Keywords:** PDZ domain, type II sodium-dependent phosphate cotransporter (NPT2A), parathyroid hormone (PTH), phosphate transport, PDZ-ligand interaction, binding affinity, phosphorylation

## Abstract

Na^+^/H^+^ exchange factor-1 (NHERF1), a multidomain PDZ scaffolding phosphoprotein, is required for the type II sodium-dependent phosphate cotransporter (NPT2A)-mediated renal phosphate absorption. Both PDZ1 and PDZ2 domains are involved in NPT2A-dependent phosphate uptake. Though harboring identical core-binding motifs, PDZ1 and PDZ2 play entirely different roles in hormone-regulated phosphate transport. PDZ1 is required for the interaction with the C-terminal PDZ-binding sequence of NPT2A (-TRL). Remarkably, phosphocycling at Ser^290^ distant from PDZ1, the penultimate step for both parathyroid hormone (PTH) and fibroblast growth factor-23 (FGF23) regulation, controls the association between NHERF1 and NPT2A. PDZ2 interacts with the C-terminal PDZ-recognition motif (-TRL) of G Protein-coupled Receptor Kinase 6A (GRK6A), and that promotes phosphorylation of Ser^290^. The compelling biological puzzle is how PDZ1 and PDZ2 with identical GYGF core-binding motifs specifically recognize distinct binding partners. Binding determinants distinct from the canonical PDZ-ligand interactions and located “outside the box” explain PDZ domain specificity. Phosphorylation of NHERF1 by diverse kinases and associated conformational changes in NHERF1 add more complexity to PDZ-binding diversity.

## 1. Introduction

**Figure 1:**
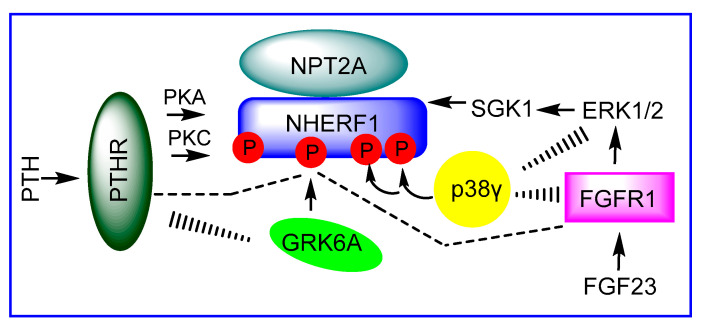
NHERF1 brings together different classes of protein for NPT2A-dependent hormone-regulated phosphate transport in kidney cells. PTH and FGF23 hormones work through PTHR and FGFR1, respectively, and activate two distinct phosphorylation pathways leading to phosphorylation of NHERF1 by diverse kinases.

Protein–protein interactions play a major role in regulation of intracellular phosphate bringing together different classes of proteins and stabilizing multiprotein complexes. The tight interaction between the type II sodium-dependent phosphate cotransporter (NPT2A) mediated renal phosphate uptake and Na^+^/H^+^ exchange regulatory factor-1 (NHERF1) is required for renal phosphate uptake. Two hormones parathyroid hormone (PTH) and Fibroblast growth factor 23 (FGF23) regulate phosphate homeostasis by controlling the NPT2A-NHERF1 association. Remarkably, PTH and FGF23 belong to different classes of receptors, a Family B G protein coupled receptor (PTHR, parathyroid hormone receptor) and receptor tyrosine kinase (FGFR1, fibroblast growth factor receptor 1), respectively, and stimulate two distinct signaling pathways involving activation of diverse kinases (Figure 1). The precise mechanism and modulators involved in NHERF1-dependent NPT2A-mediated hormone-sensitive phosphate uptake are not completely understood.

**Figure 2:**
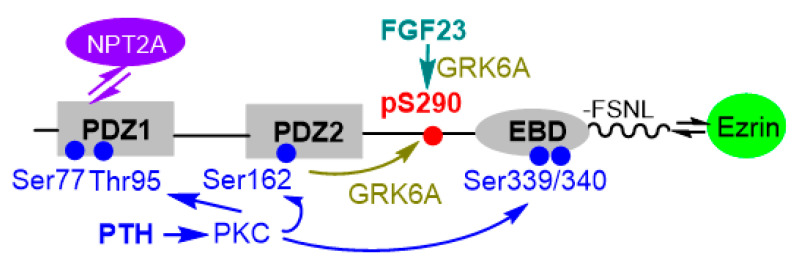
Linear representation of NHERF1. NPT2A-dependent PTH- and FGF23-sensitive phosphate transport converge on Ser^290^ of NHERF1. PTH-stimulated phosphorylation employs PKC/PKCα. Both processes cause conformational changes in NHERF1 allowing GRK6A to phosphorylate Ser^290^ and disengage NPT2A from NHERF1-Ezrin complex, thereby arresting phosphate transport.

NHERF1 (EBP50) belonging to the NHERF family [1] of PDZ adaptor proteins controls protein localization at the apical plasma membrane of polarized epithelial cells [2,3]. NHERF1 tethers binding partners through tandem PDZ domains named for the common structural domain shared by the postsynaptic density protein (PSD95), Drosophila disc large tumor suppressor (DlgA), and zonula occludens-1 protein (ZO-1), and a C-terminal ezrin-binding domain (EBD) associated with ezrin, radixin, and moesin through extreme C-terminal end representing a PDZ-ligand motif itself (-FSNL) (Figure 2). [4]. PDZ domains are approximately 90 aa in length and commonly represented as a box (Figure 2). Two flexible unstructured linkers connect PDZ1 and PDZ2, and PDZ2 and EBD. PDZ-dependent binding partners of NHERF1 include G-protein couple receptors (GPCR’s) (PTHR, CXCR2, β2AR), ion transporters (NPT2A, CFTR), transcriptional modifiers (TAZ/YAP65), and phosphoenzymes and posttranslational regulators (PKCα, GRK6A, SGK1, RGS14, MAPKs) (Table 1). These proteins may selectively bind PDZ1 or PDZ2, or both PDZ domains. The regulation of renal proximal tubular phosphate reabsorption requires PDZ-dependent interactions between NHERF1, NPT2A, and GRK6A.

NPT2A (*SLC34A1*), the primary renal Na-dependent phosphate transporter, mediates hormone-regulated phosphate transport and is an essential binding partner of NHERF1 [4,5,6,7,8]. NPT2A has eight putative transmembrane domains and several intra- and extracellular loops. The NPT2A intracellular C-terminus ends with a carboxy-terminal PDZ motif (-T^−2-^R-L^0^) that mediates its binding to NHERF1. Loss-of-function mutations in NHERF1 (PDZ1/L^110^V, PDZ2/R^153^Q and E^225^K) or NPT2A (R^495^H/C and S^585^P) disrupt phosphate metabolism and lead to hypophosphatemia [9,10,11,12]. Parathyroid hormone (PTH) and fibroblast growth factor 23 (FGF23) dissociate the NPT2A-NHERF1 binary complex by activating two distinct signaling pathways that converge at NHERF1 [13,14,15,16] resulting in NPT2A internalization and downregulation. PTH works through its cognate parathyroid hormone receptor (PTHR). PTHR contains seven transmembrane domains linked by three extracellular and three intracellular loops, and the intracellular C-terminus has a PDZ-binding sequence (-E^−3^-T^−2^-V-M^0^). PDZ-ligand interaction between NHERF1 and PTHR is critical for receptor signaling [17,18]. It is thought that NHERF1 homodimerization may play a key role in the formation of an extended multiprotein signaling complex that includes NHERF1, NPT2A, ezrin, PTHR and some kinases [17,19,20,21,22]. The PDZ domains in NHERF1, like other such structures, are relatively rigid units that were crystallized and characterized [23,24]. Linkers between PDZ1 and PDZ2 and between PDZ2 and EBD are unstructured and flexible. This contributes to the challenge of solving the structure of full-length NHERF1. Both NHERF1 PDZ domains have a classical PDZ fold with two alpha-helixes and six beta-sheets and share 65% identity or sequence similarity. PDZ1 and PDZ2 are Class I PDZ domains with identical GYGF core-binding motifs, known also as carboxylate-binding sites. Available X-ray structures of PDZ1 or PDZ2 bound to the C-terminus of β2-AR (-DSLL), cystic fibrosis transmembrane conductance regulator (CFTR) (-DTRL) [23,24], or chemokine receptor-2 (CXCR2) (-STTL) [25,26] demonstrate that the preserved C-terminal X-S^−2^/T-X-Φ^0^ motif of peptide ligands (PDZ-binding motif) occupies the PDZ domain cavity as an antiparallel β-sheet. By convention, the sequential residues forming a PDZ-binding motif are numbered starting from the last position (Φ ^0^) and going upstream toward the N-terminus as –1, –2, –3, etc. Φ^0^ forms hydrophobic interactions with the GYGF motif, as well as hydrogen bonds through backbone atoms of PDZ1/PDZ2. Ser^−2^/Thr^−2^ of target C-terminal PDZ ligands establishes a direct side chain hydrogen bond with His^72^ or His^212^ from the top of the α2 helix of PDZ1 and PDZ2, respectively. These interactions are conserved, called canonical, and characterize Class I PDZ domains [23,24,27]. Mutation of Tyr^24^/Tyr^164^ and Phe^26^/Phe^166^ to Ala in the GlyTyrGlyPhe [GYGF hereafter] motif (GlyAlaGlyAla sequence [GAGA hereafter]) of PDZ1/PDZ2 disrupts the hydrophobic network and blocks the association with ligands [4,28]. Remarkably, a substitution of the conserved Thr^−2^ by Cys in the C-terminus of PTHR (-ETVM) decreases but does not eliminate binding with His^212^ of PDZ2 and more essentially does not interfere with PTHR function [29]. 

In addition to canonical interactions, NHERF1 PDZ1 and PDZ2 also form non-canonical interactions distal from the PDZ binding groove and located “outside the box”. The presence of such noncanonical interactions is illustrated by various domain-selective binding of different proteins that selectively engage NHERF1 PDZ1 or PDZ2 despite the identical canonical binding sites (Table 1). The non-canonical binding determinants are unique for individual PDZ domains [30]. There are several typical features making “outside the box” interactions specific. Frequently these interactions have an electrostatic nature [31]; some of them are regulated by phosphorylation [32,33,34] or involved in a long-range allosteric network [35]. Further, the helical-turn-helical (ɑ3-turn-ɑ4) extension is an essential structural element common for many PDZ domains and represents a universal element stabilizing PDZ-ligand binding [36,37]. Whether the ɑ3-loop-ɑ4 extension allosterically affects the binding with target ligands or directly contacts upstream N-terminal residues of the bound target remains to be established.

The main feature making NHERF1 unique is its C-terminal tail corresponding to a PDZ-ligand motif (-FS^−2^NL^0^). It is believed that -FS^−2^NL^0^ may occupy PDZ2 rather than PDZ1 to form a self-inhibited conformation [38]. The physiological role of the self-inhibited conformer remains to be elucidated. The putative ability of NHERF1 to dimerize through its C-terminal PDZ-motif (-FS^−2^NL^0^) was extensively investigated [26,39,40,41,42]. Based on dynamic light scattering experiments, NHERF1 in solution is monodispersed [43]. Curiously, the NHERF1-NHERF1 association decreased in the presence of okadaic acid suggesting that phosphorylation may regulate dimerization [40]. A comparable effect was observed in the presence of the C-terminal PDZ-ligand motif of the β2-AR (-DSLL) [41]. The biological importance of NHERF1 dimerization may relate to its ability to assemble large multi-protein complexes and serve as an adapter for different classes of proteins. However, this theory has not been analyzed in detail.

NHERF1 is a phosphoprotein harboring 31 Ser and 9 Thr residues. Notably, there is a high-density Ser cluster in the flexible hinge region of NHERF1 linking PDZ2 and the EBD with 17 Ser/Thr residues in this segment. Ser^290^ located in this region was identified as a phosphorylation site that allosterically regulates the interaction between NHERF1 and NPT2A through conformational changes near Glu^43^ [44], a site that determines the binding specificity between PDZ1 and NPT2A [45]. Furthermore, dephosphorylation-phosphorylation cycling of Ser^290^ regulates NHERF1 self-assembly and NPT2A-dependent hormone-sensitive phosphate transport [44]. Remarkably, both PTH and FGF23 pathways contribute equally to the stabilization of the open state of NHERF1 via phosphorylation of Ser^290^ by G Protein-coupled Receptor Kinase 6A (GRK6A) (Figure 1) [46]. PTH-induced phosphorylation of Ser^77^ in conjunction with Thr^95^ has a similar effect on regulating phosphate uptake as does Ser^290^ phosphorylation. Ser^77^ and Thr^95^, located in PDZ1, are predicted PKC phosphorylation sites (Figure 1) [47,48]. Ser^162^ is a defined PKCɑ phosphorylation site [43,49]. Notably, PKCɑ is a major pathway of PTH signaling [28,50,51] and is the only PKC isoform with a PDZ-recognition motif at its C-terminus (-SAV^672^) [52,53]. Two other PKC phosphorylation sites, Ser^339^ and Ser^340^, in the C-terminal region (Figure 2), promote conformational reorganization in NHERF1 and facilitate phosphorylation of Ser^162^ [43]. The role of this remarkable cooperativity on hormone-induced phosphorylation of NHERF1 has not yet been examined in vivo. Whether additional phospho-Ser/Thr sites regulate NHERF1-ligand interactions, conformational diversity, or phosphate transport is not known.

## 2. NHERF1 (PDZ1) Specifically Binds NPT2A for Hormone-Sensitive Phosphate Transport

### 2.1. NHERF1 PDZ1 and PDZ2 Domains Are Not Interchangeable

The association between NHERF1 PDZ1 and its natural ligand NPT2A (*SLC34A1*) is a perfect example representing NHERF1 PDZ-ligand specificity. The NHERF1 PDZ1-NPT2A complex is required for NPT2A-mediated hormone-sensitive renal phosphate transport [15,54,55]. The interaction between PDZ1 and NPT2A occurs through the PDZ-ligand C-terminal -T^−2^R^-1^L^0(639)^ sequence of NPT2A. The PDZ1-NPT2A complex is not assembled in the presence of NPT2A-Leu^639^Ala, a variant that has a defective C-terminal PDZ recognition motif (-T^−2^R^−1^L^0^/A) [4]. PDZ1 interacts with the C-terminal peptide ligand of NPT2A within the low micromolar range (3–5 µM), whereas the association between PDZ2 and NPT2A is insignificant [4,45]. The biological enigma is why and how PDZ1 and PDZ2 with identical conserved binding sites (GYGF and His) interact uniquely with NPT2A PDZ1. Analysis of possible binding determinants “outside the box” pointed to Glu^43^ located in the α1 helix of PDZ1. MD simulations applied in our study predict the formation of an ionic pair between Glu^43^ of PDZ1 and Arg^−1^ of the NPT2A C-terminal -T^−2^R^−1^L^0^ motif [45]. Long-range MD simulations (~ 100-ns) provide clear evidence that the negatively charged side chain of Glu^43^ and the side chain of His^27^ are involved in electrostatic interactions with the positively charged side chain of Arg^−1^ of NPT2A (-T^−2-^R^−1-^L^0^) and, more importantly, these interactions are persistent on the MD simulation time scale. In contrast to PDZ1, PDZ2 possesses Asp^183^ and Asn^167^ at the homologous positions. However, these residues do not form a stable interaction with Arg^−1^ [45]. We propose that the side chain of Asp^183^, which is relatively short compared to Glu^43^, is thereby unable to support direct interaction with the side chain of Arg^−1^. Our MD simulations [45] and available NMR structure [36] demonstrate that the sidechain of Asp^183^ is flexible and not involved in a stable interaction with Arg^−1^ and provide provisional support of this theory. Notably, the Glu^43^Asp mutation in PDZ1 leads to dramatic loss of affinity with a similar C-terminal -TRL motif of CFTR [36], another biological partner of NHERF1 [56]. CFTR, like NPT2A, largely associates with PDZ1 but not with PDZ2 [57,58,59]. Recently published X-ray structures and MD simulations of the PDZ2-CFTR complex suggest that the interaction between Asp^183^ and Arg^−1^ is underestimated and can be an important element of the complex [60]. However, how the association between Asp^183^ and Arg^−1^ regulates binding affinity between PDZ2 and CFTR remains unresolved.

**Figure 3:**
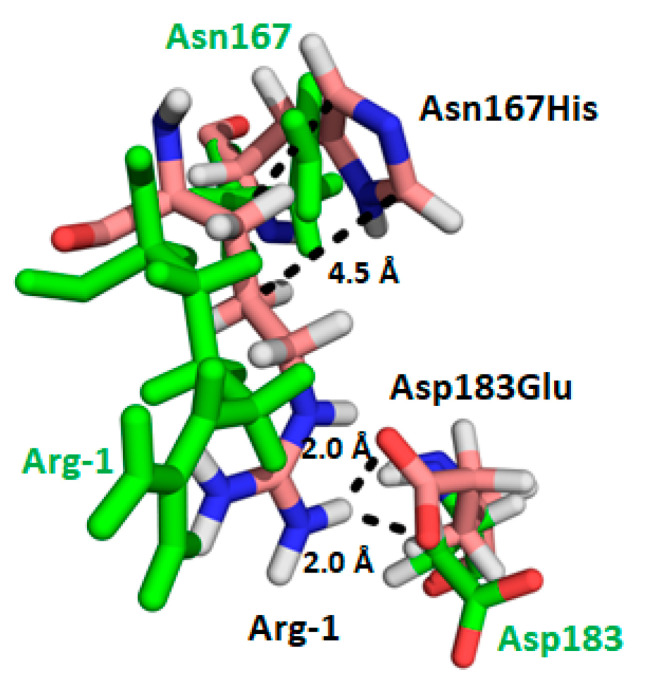
Double mutation of Asp^183^ to Glu and Asn^167^ to His in PDZ2 favors the formation of electrostatic interactions with Arg^-1^ of the C-terminal -TRL motif of NPT2A shown in wheat sticks. The predicted interactions stabilizing the complex are shown as black dotted lines. Wild-type Asp^183^ and Asn^167^ of PDZ2 shown in green sticks do not interact with Arg^-1^ of NPT2A (green sticks) [45].

**Figure 4:**
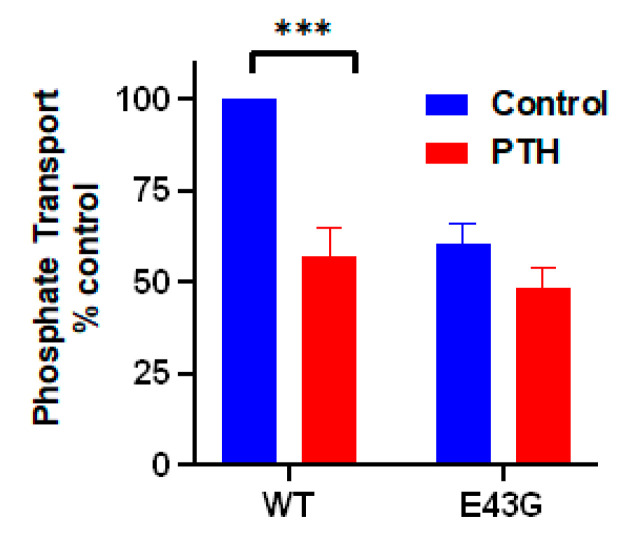
Mutation of Glu^43^ to Gly in PDZ1 of NHERF1 inhibits NPT2A-dependent PTH-sensitive phosphate transport. Results report the mean ± SEM (*n* = 3, *** *p* < 0.001, ANOVA) [44].

**Figure 5:**
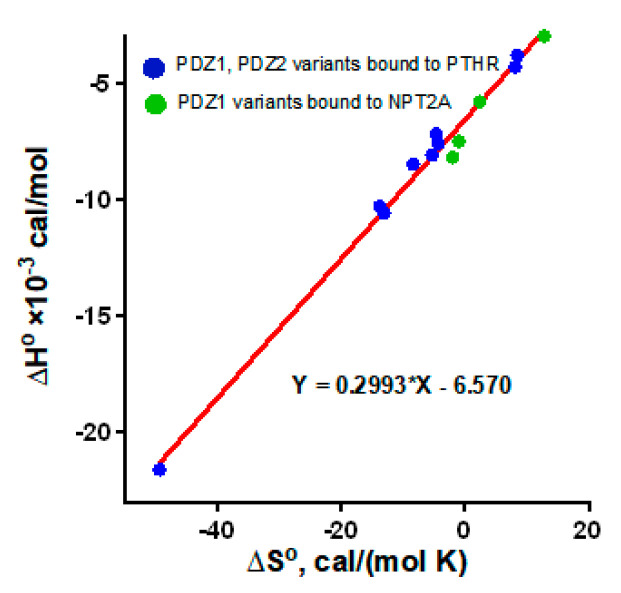
Linear regression of ∆H^ο^ vs. ∆S^ο^. Data for wild-type PDZ1, PDZ1-His^27^Asn, PDZ1-Glu^43^Asp, and PDZ1-His^27^Asn/Glu^43^Asp bound to the C-terminal motif of NPT2A are green (data from Table 2 [45]). Data for PDZ1 and PDZ2 bound to the ensemble of Ala variants of the C-terminal motif of PTHR are blue (data from Table 4 [29]).

To explore specificity of Glu^43^ and His^27^ on the interaction between PDZ1 and NPT2A we generated a PDZ1 variant where Glu^43^ and His^27^ were replaced by Asp and Asn, respectively. As anticipated, PDZ1 with the Glu^43^Asp/His^27^Asn mutations eliminates binding between PDZ1 and NPT2A. The relevance of Glu^43^ and His^27^ on NPT2A-dependent PTH-sensitive phosphate transport was validated by measuring phosphate uptake in OKH cells expressing the Glu^43^Asp/His^27^Asn-NHERF1 variant. As we presumed, Glu^43^Asp/His^27^Asn-NHERF1 blocks basal phosphate transport and is refractory to PTH [44]. Notably, when Asp^183^ and Asn^167^ in PDZ2 were replaced respectively by Glu and His, the corresponding residues in PDZ1, the Asp^183^Glu/Asn^167^His rescue variant bound the NPT2A C-terminal -TRL motif (Figure 3) [45]. However, Asp^183^Glu/Asn^167^His-NHERF1 did not support NPT2A-dependent PTH-sensitive phosphate uptake (unpublished observations). As expected, mutation of Glu^43^ to Gly in PDZ1 of NHERF1 blocked phosphate uptake (Figure 4) [44]. Experimental study confirms that PDZ1 is required for formation of the NPT2A complex, and regulation of NPT2A-mediated hormone-sensitive phosphate transport. This finding strongly supports the concept that the specificity of the PDZ1 domain is not determined by the conserved subset of residues (-^23^GYGF^26^- and His^72^) but rather by the “outside the box” determinant Glu^43^. Support for this conclusion also comes from thermodynamic parameters determined by isothermal microcalorimetry [45]. Substitution of Glu^43^ by Asp increases enthalpy (Δ*H*^ο^) and provides a jump in entropy (Δ*S*^ο^) making the interaction unfavorable. Mutation of His^27^ to Asn has a minor effect. A small change in the free energy (ΔΔG^ο^), 0.8 and 0.4 kcal/mol for Glu^43^→Asp and His^27^→Asn, respectively, is attributed to the enthalpy-entropy compensation [45] illustrated in Figure 5.

### 2.2. The Role of the NPT2A Internal -TRL- Motif Remains to Be Explored

**Figure 6:**
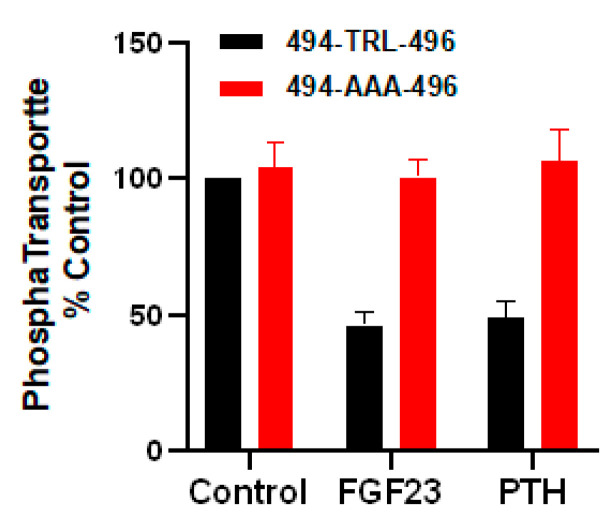
Mutation of the WT internal PDZ-binding 494-TRL-496 motif of NPT2A to 494-AAA-496 blocks PTH- and FGF23-sensitive phosphate uptake. PTH- and FGF23-inhibitable phosphate uptake was measured in NPT2A CRISPR/Cas9 knockout OK cells transfected with a human NPT2A construct with carboxy-terminal -T^−2^R^-1^L^0^ → -AAA substitution. Results report the mean ± SEM (*n* = 5, *p* < 0.0001, ANOVA) (unpublished observation).

In addition to its canonical C-terminal -T^−2^RL^0^ PDZ-binding motif, NPT2A possess an internal PDZ -^494^T^−2^RL^0^- recognition sequence that has not been characterized. The C-terminal motif is critical for interaction with NHERF1 [4]. Whether both PDZ motifs contribute to or are required for proper localization and function of NPT2A and hormone action is unknown. Two disease-associated mutations (Arg^495^His, Arg^495^Cys) have recently been described in the putative internal PDZ motif (-^494^TRL^496^-), while a third (Ser^585^Pro) is located at the carboxy-terminal region. All are associated with elevated renal phosphate excretion and consequent hypophosphatemia [10,11,61]. NPT2A-Arg^495^His and NPT2A-Ser^585^Pro display different cell localization compared to wild-type (WT) NPT2A [61]. The carboxy-terminal PDZ-binding motif (-T^−2^R^-1^L^0^) [4,45,62] is required for cell membrane NPT2A localization and is necessary for hormone-regulated phosphate transport [4,6]. The involvement of the putative internal NPT2A PDZ motif in association with NHERF1 and its role in hormone action is not described. Pilot results indicate that mutation of the internal PDZ motif interferes with PTH and FGF23 action and inhibits regulated uptake (Figure 6) despite normal binding to NHERF1 (unpublished work).

## 3. NPT2A-Dependent Hormone-Inhibitable Phosphate Transport Requires Association between PDZ2 and GRK6A

**Figure 7:**
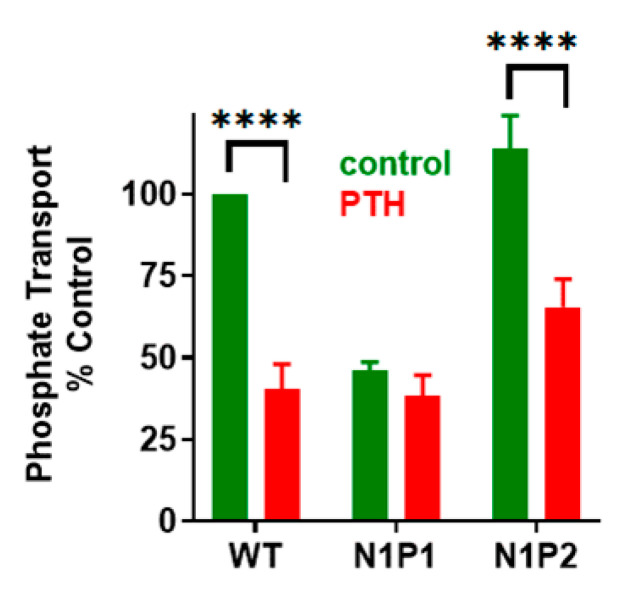
Effect of NHERF1 PDZ mutants on basal and PTH-sensitive phosphate uptake. OKH cells were transiently transfected with WT-NHERF1, N1P1-GAGA, or N1P2-GAGA. Cells were treated with vehicle or with 100 nM PTH(1-34). N1P2-GAGA-NHERF1 and WT-NHERF1 support PTH-inhibitable phosphate uptake, whereas N1P1-GAGA does not. Results report the mean ± SEM (*n* = 4, **** *p* < 0.0001, ANOVA) [63].

**Figure 8:**
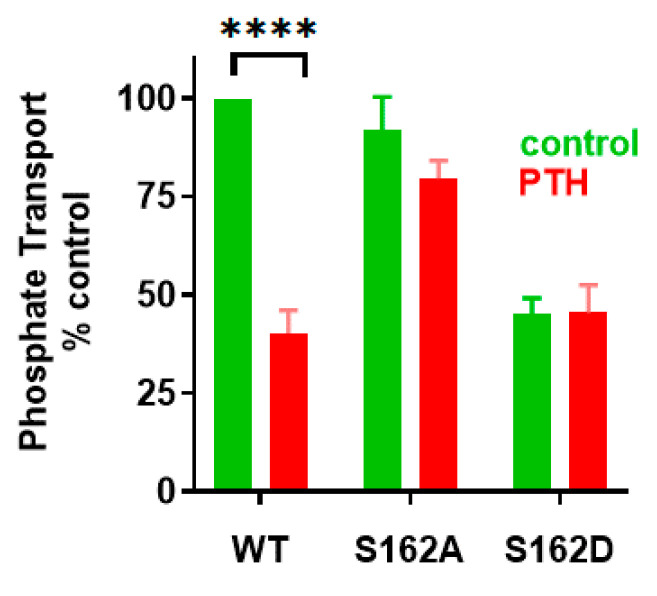
Ser^162^ is essential for PTH-inhibitable phosphate uptake. OKH cells were transiently transfected with WT-NHERF1 or with Ser^162^Ala-NHERF1 or Ser^162^Asp- NHERF1. Cells were treated with vehicle or with 100 nM PTH(1-34). Results report the mean ± SEM (*n* = 4, **** *p* < 0.0001, ANOVA) [63].

G protein-coupled receptor kinase 6A (GRK6A), a natural partner of NHERF1, possesses a canonical PDZ ligand (-T^−2^RL^0^) at its C-terminus. GRK6A, like NPT2A, associates with NHERF1 PDZ domains through its C-terminal motif (-T^−2^R^−1^L^0^). Knocking down Grk6a by siRNA blocks Npt2a-dependent phosphate uptake in response to PTH [63]. Thus, GRK6A is an essential regulatory component of NPT2A-dependent PTH-sensitive phosphate transport and corroborates previous findings that GRK6A pharmacological inhibitors abolish PTH action [44]. Binding affinities (3–5 µM) for the C-terminal PDZ ligand of GRK6A [63] or NPT2A (22 aa) [45] with NHERF1 are comparable and suggest that the binding mechanism is presumable identical. NPT2A and GRK6A interact with PDZ1 with a greater affinity than to PDZ2, thereby confirming that PDZ1 is naturally optimized to bind the -TRL sequence. Intriguingly, a minor interaction between GRK6A and PDZ2 is nonetheless critical for constitutive or PTH-induced phosphorylation of NHERF1 at Ser^290^ [44,46], and for PTH-sensitive phosphate transport [44]. This finding highlights a biological puzzle as to how PDZ2 becomes accessible to GRK6A and the role of PTH and FGF23 in this process. Again, canonical PDZ recognition sites (-GYGF- and His) required for the binding of the C-terminal Leu^0^ and Thr^−2^ of GRK6A cannot explain the observed specificity for PDZ2. Furthermore, mutation of the NHERF1 PDZ2 core-binding GYGF motif (N1P2-GAGA) decreased but did not abolish phosphate transport in response to PTH (Figure 7). This finding strongly suggests that the PDZ2 domain retains its ability to interact with the C-terminus of GRK6A in vivo. Clearly, binding determinants “outside the box” control the formation of the PDZ2-GRK6A complex. Analysis of residues that may contribute to the binding pointed to Ser^162^, known as a PKCα phosphorylation site in human NHERF1 [43,49]. Notably, NHERF1 homologs (mouse, rabbit) harbor Asn at the corresponding position. PKCα action is unique for PDZ2 inasmuch as PDZ1 has Asn^22^ at the homologous location. The published docking structure of PDZ2 interacting with its cognate C-terminal peptide demonstrates that Ser^162^ is masked by the C-terminal tail [43]. The in vivo significance of post-translational modification of Ser^162^ is unknown. We initiated studies to elucidate the role of NHERF1 Ser^162^ on NPT2A-dependent phosphate transport. Ala replacement at Ser^162^ (NHERF1 Ser^162^Ala) diminished PTH-inhibitable phosphate transport suggesting that Ser^162^ is an essential regulator of hormone-sensitive phosphate uptake. Unexpectedly, phosphomimic Ser^162^Asp mutation in NHERF1 disrupts basal phosphate transport and blocks PTH action on phosphate uptake (Figure 8) [63]. Clearly, the discrepancy in charge and size between phosphate and carboxylate groups of phosphorylated Ser and Asp, respectively explains the observed difference. Since the phosphomimic mutation is structurally remote and cannot directly mediate the interaction between PDZ1 and NPT2A we assume that NHERF1-Ser^162^Asp exerts a large conformational change that affects the interaction between PDZ1 and NPT2A. There is no high-resolution structure of full-length NHERF1. We speculate that incorporation of Asp^162^ with the negatively charge carboxylate group interrupts the self-inhibited NHERF1 conformation [38], releases the C-terminal tail, and increases conformational dynamics of NHERF1. The structurally disordered C-terminal tail may adopt a variety of conformations and screen the association between PDZ1 and NPT2A. Another possibility is that the C-terminal tail engages PDZ1 and interferes with NPT2A binding. In contrast to NHERF1-Ser^162^Asp, the double phosphomimic mutation at Ser^339^Asp/Ser^340^Asp increases the binding affinity of both PDZ1 and PDZ2 for CFTR through conformational changes in the linker regions [43] and long-range allosteric cooperativity in NHERF1 [36]. Of note, Ser^339^/Ser^340^ are PKC phosphorylation sites [43,49] like Ser^162^ [46]. It was suggested that Ser^339^/Ser^340^ phosphorylation promotes phosphorylation of Ser^162^ [43]. Whether this cooperativity exists in vivo remains to be established.

**Figure 9:**
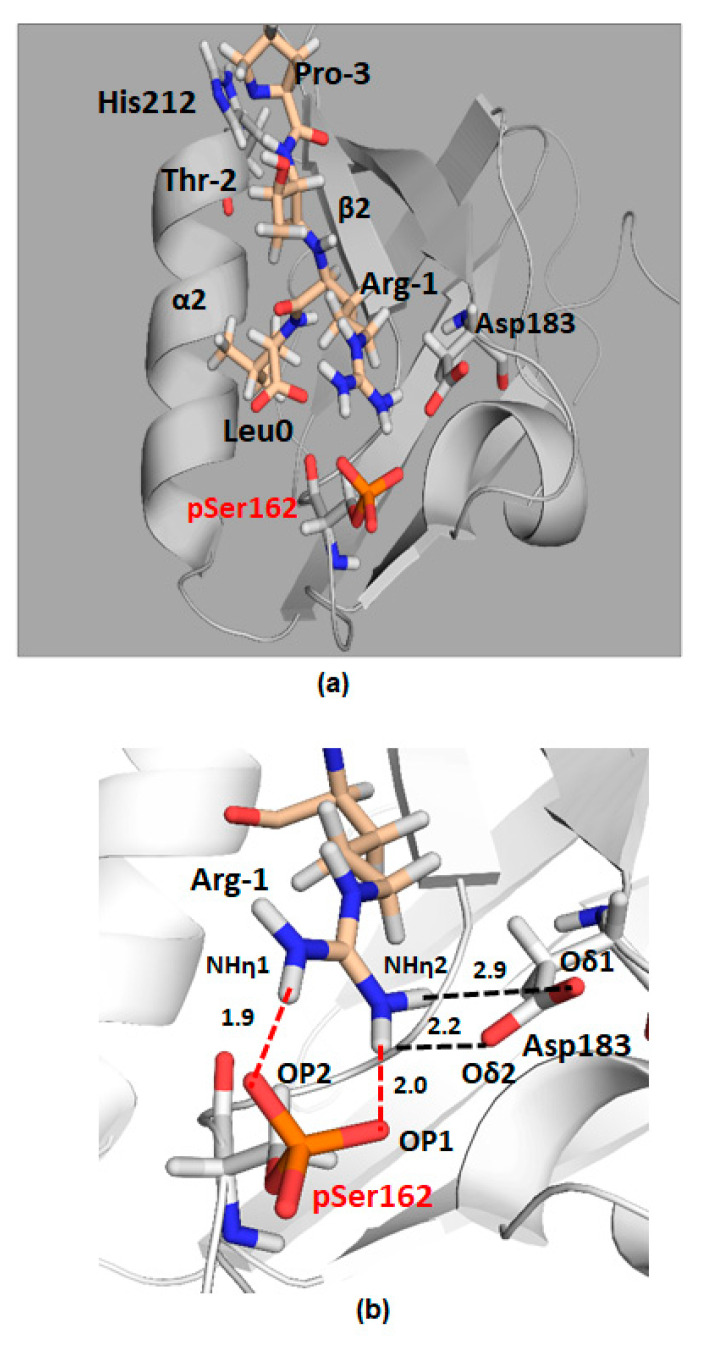
Computational model of pSer^162^-PDZ2 bound to the C-terminal peptide of GRK6A. The PDZ2 domain is highlighted in grey and the GRK6A peptide is represented in wheat sticks. (**a**), The extreme C-terminus of GRK6A (-PTRL) is inserted in the binding pocket of pSer^162^-PDZ2 between the α2 helix and the β2 sheet. (**b**), electrostatic interactions between pSer^162^ or Asp^183^ of PDZ2 and Arg^−1^ of GRK6A are shown as red and black dotted lines, respectively; Average distances between OP1 of pSer^162^ and NHη^1^ of Arg^−1^ or between OP2 and NHη^2^ are 2.0 Å and 1.9 Å, respectively; between NHη^1^ of Arg^−1^ and Oδ^1^ or Oδ^2^ of Asp^183^ are 2.9 Å and 2.2 Å, respectively. Distances were calculated along the last 10-ns of MD simulation. Hydrogen atoms are white, oxygens are red, and nitrogens are blue.

To explore the biochemical role of phospho-Ser^162^ (pSer) and its impact on the interaction between NHERF1 PDZ2 and GRK6A, pSer^162^ was genetically introduced in recombinant PDZ2 (133-300 aa) using amber codon suppression [64]. Previously, semi-synthesis was effectively applied to generate site-specific phosphorylated PDZ domains [33]. Here, we used amber codon suppression to genetically encode pSer at position 162 [63], which in the future will allow to generate phosphorylated full-length proteins comprising both PDZ domains. Replacement of Ser^162^ by pSer^162^ in recombinant PDZ2 permits estimating the binding affinity between pSer^162^-PDZ2 and the C-terminal PDZ-ligand of GRK6A. Fluorescence anisotropy (FA) was applied to measure dissociation constants (*K*_D_’s) between pSer^162^-PDZ2, Ser^162^Ala-PDZ2, wild type PDZ2 (133–300 aa) and FITC-labeled GRK6A (22 aa). The results demonstrate that the *K*_D_ values for the interaction between WT PDZ2 or Ser^162^Ala-PDZ2 and GRK6A were comparable (51.3 ± 0.4, 43.4 ± 0.4 µM) and double that for pSer^162^-PDZ2 (26.1 ± 0.8 µM), thus, demonstrating that the binding affinity of PDZ2 is regulated by phosphorylation of Ser^162^. Site-specific incorporation of pSer^162^ applied for NHERF1 PDZ2 introduces an analogous but not identical functional group compared to phosphomimetic mutagenesis. The phosphate group has a –2 negative charge compared to the single negative charge of the Asp carboxylate group. The increased size of the sidechain may importantly perturb the local protein conformation [65]. MD simulations provide structural details associated with incorporation of pSer^162^ (Figure 9). Three main observations come from the simulation studies. First, pSer^162^ forms an electrostatic interaction with the positively charged side chain of Arg^−1^ of the C-terminal TRL motif of GRK6A and therefore has a significant impact on dynamics and conformational flexibility of the C-terminus of GRK6A. Second, pSer^162^ promotes the formation of the electrostatic network (pSer^162^- Arg^−1^-Asp^183^) wherein the sidechain of Asp^183^ changes its orientation and moves toward the sidechain of Arg^−1^. Third, the GYGF/GAGA substitution in the carboxylate-binding site of pSer^162-^PDZ2 does not impede the interaction with GRK6A. Consequently, NHERF1 with the modified core-binding motif (N1P2-GAGA) supports PTH-sensitive phosphate uptake (Figure 7). Thus, a strong stabilizing influence of pSer^162^ underscores the limitation and potential hazard of using phosphomimetics to draw conclusions about phosphorylation and demonstrates the strength of introducing site-specific pSer using experimental and computational methods.

## 4. Conformational Reorganization of NHERF1 Regulates NHERF1-NPT2A Interactions

### 4.1. Phosphorylation of Ser290 Controls NHERF1 Conformation and Interactions with NPT2A

Recent combined NMR and small-angle neutron scattering (SANS) experiments revealed that full-length NHERF1 cannot be characterized by a single conformation. Rather, NHERF1 represents an ensemble of diverse PDZ configurations connected by flexible linkers with the C-terminal unstructured tail [66]. Notably, the flexible linker connecting PDZ2 and the C-terminus has 17 Ser/Thr residues. Compared to structurally determined rigid domains, intrinsically disordered regions typically contain a high density of phosphorylation sites [67]. Site-specific phosphorylation within these regions promotes structurally relevant conformational transitions that affect protein function [67,68]. Reversible post-translational modification of Ser/Thr residues within this region may regulate NHERF1 activity and signaling. We showed that dephosphorylation-phosphorylation of Ser^290^ located in this flexible linker regulates the association between NHERF1 PDZ1 and NPT2A. Preventing Ser^290^ phosphorylation with a phosphoresistant mutation (Ser^290^Ala) or pharmacologically inhibiting the action of GRK6A kinase decreases the binding of NTP2A to NHERF1 and reduces PTH-sensitive phosphate transport [44]. Evidently, Ser^290^ determines regulated stability of the NHERF1-NPT2A complex through long range allosteric communication. Hydrogen deuterium exchange mass spectrometry (HDX-MS) analysis was used to analyze the region in NHERF1 undergoing conformational changes along the dephosphorylation-phosphorylation cycle of Ser^290^ [44]. In addition to the linker region between PDZ2 and EBD, substantial conformational changes were found in PDZ1 near Glu^43^, a critical determinant for NPT2A binding [44,45]. Thus, dephosphorylation-phosphorylation of Ser^290^ allosterically regulates the conformation of the side chain of Glu^43^ and switches the interaction on and off with Arg^−1^ of the C-terminal -TRL motif of NPT2A and thereby controls PTH-sensitive phosphate uptake.

### 4.2. NHERF1 Disease-Associated Mutations (Leu^110^Val, Arg^153^Gln and Glu^225^Lys) Affect Conformational Landscape and Interaction with NPT2A

Another example of allosteric regulation within NHERF1 relates to Leu^110^Val, Glu^225^Lys and Arg^153^Gln mutations identified in patients with hypophosphatemia [9]. The disease-associated mutations decrease or abolish the interaction between PDZ1 and NPT2A and block PTH-sensitive phosphate transport [4]. It was suggested that Arg^153^Gln and Glu^225^Lys, located in PDZ2, stabilize a self-inhibited conformation in which the NHERF1 C-terminus -FS^−2^NL^0^, a PDZ recognition motif itself, is engaged in the PDZ2 domain, masking PDZ1 and thereby disrupting the interaction with NPT2A [4]. Remarkably, when the C-terminal Leu^0^ of the NHERF1 PDZ ligand, required for engagement with PDZ2, was exchanged for Ala (NHERF1 Leu^0^Ala) and was paired with the Arg^153^Gln (NHERF1 Arg^153^Gln/Leu^358^Ala) disease mutation, the interaction with NPT2A was rescued and PTH-sensitive phosphate transport was restored and indistinguishable from that of wild-type NHERF1 [4], notably in the ongoing presence of the naturally occurring Arg^153^Gln mutation. Leu^110^Val, a similar dysfunctional mutant, locates in the α4 helix of the carboxy-terminal helix-turn-helix extension (α3-loop-α4) of PDZ1 [36]. This subdomain of 30 residues allosterically regulates the binding affinity between NHERF1 PDZ domains and their cellular targets [69]. NMR studies suggested that an extensive hydrophobic network between PDZ1 and the α3-loop-α4 extension stabilizes the structure with Leu^110^ serving as a part of this hydrophobic network (64). It was proposed that mutation of Leu^110^ to Val could potentially disrupt the packing interactions and reduce the stability and affinity of the PDZ1 domain [36]. Replacement of Leu^110^, even with a shorter hydrophobic side chain of Val, will likely disturb the hydrophobic network inside the α3-loop-α4 extension and promote its rearrangement. In addition, conformational changes associated with such an Leu^110^Val mutation may limit phosphorylation of Thr^95^, a PKC phosphorylation site located near Leu^110^, and thereby disrupt cooperativity between Thr^95^ and Ser^77^ phosphorylation events required for normal NPT2A-dependent PTH-inhibitable phosphate transport [70]. These conjectures require experimental verification.

## 5. “Outside the Box” Determinants Are Involved in the Interaction between NHERF1 PDZ Domains and PTHR

PTHR, a Family B GPCR, plays a key role in mineral-ion metabolism and bone physiology [2,22]. The C-terminal sequence of PTHR represents a typical PDZ binding motif (-T^−2^-V-M^0^) with upstream Glu residues at positions -3, -5, and -6 (-E^−6^-E^−5^-W-E^−3^-T^−2^-V-M^0^). Although crystallographic or NMR structures for the complex between the PDZ domains of NHERF1 and PTHR have not been solved, molecular determinants of the interaction were characterized by different approaches. Biochemical studies [18,71] and Molecular Dynamics simulations [62] predict that both PDZ domains engage in PDZ-ligand interactions with C-terminal Met^0^ and Thr^−2^ of PTHR. The promiscuous -T^−2^-V-M^0^ motif permits PTHR to bind PDZ1 or PDZ2, or both through canonical PDZ-ligand interactions established between the conserved GYGF motif of PDZ1/PDZ2 and the His^72^/His^212^ residue at the top of the α2 helix. Although these canonical interactions are essential components of the binding, determinants outside the PDZ-ligand pocket enhance formation of the binary [NHERF1-PTHR] complex [18,29]. MD simulations on a ~70-ns time scale provide clear evidence that Arg^40^ and His^27^ or Arg^180^ of PDZ1 and Asn^169^ in PDZ2 are involved in forming stable interactions with Glu^−3^ of PTHR [29,62]. Fluorescence anisotropy (FA), and isothermal titration calorimetry (ITC) measurements experimentally validated and extended computational predictions by showing that Glu^−3^ to Ala substitution destabilizes the complex [29]. This finding corroborates X-ray crystallographic [23,24] and NMR studies [36] that demonstrated the formation of electrostatic interactions between Arg^40^/Arg^180^ of PDZ1/PDZ2 and Asp^−3^ of the C-terminal PDZ-binding motif of CFTR (-DTRL). Similar electrostatic interactions between NHERF1 PDZ1/PDZ2 and Asp^−3^ from the C-terminal PDZ-binding motif of the β-AR (-ESLL) were confirmed by X-ray crystallography [23,24]. The determined binding affinities in the nM–µM range for PDZ1 or PDZ2 domains bound the C-terminal peptide ligands of CFTR or PTHR support the formation of favorable electrostatic interactions [29,36,43,72]. 

In addition to the Arg^40^/Arg^180^-Glu^−3^ pair, residues from the β2-β3 loop of PDZ1/PDZ2 and the negatively charged Glu residues at positions –5 and –6 of the PTHR C-terminus are also involved in the interactions [29]. The β2-β3 loop is flexible. This explains why the impact of the residues from the loop on the binding with the upstream residues of the PTHR (E^−6^E^−5^-WETVM) is challenging to estimate by MD simulation [29]. The affinities determined by a plate-binding assay demonstrate that mutation of Glu^585^ (Glu^−5^) to Ala or double substitution of Glu^585^Ala/Glu^586^Ala decreases binding to NHERF1 [18]. Thermodynamic parameters of the binding between NHERF1 PDZ domains and the C-terminal peptide of PTHR (8 aa) with a single or double Ala substitution were evaluated by ITC [29]. The loss of the binding strength following Glu-to-Ala substitution is attributed to unfavorable changes in enthalpy (Δ*H*^ο^) and entropy (Δ*S*^ο^). Mutation of Glu^−5^ in combination with Glu^−6^ exerts a synergistic effect on Δ*H*^ο^. Again, a corresponding change in the free energy of binding (ΔΔG^ο^) is not observed or is only minor due to the enthalpy-entropy compensation and rather define the PDZ-ligand specificity [29]. The ΔS^ο^ values for PDZ1 and PDZ2 domains bound to the ensemble of Ala variants of the C-terminal motif of PTHR were plotted against Δ*H*^ο^. A strong correlation between ΔS^ο^ and Δ*H*^ο^ is well-matched to PDZ1-NPT2A mutant variants (Figure 5). A linear plot for PDZ1-NPT2A and PDZ1/PDZ2-PTHR confirmed that the binding mechanism is similarly conserved. 

## 6. Concluding Remarks

NHERF1 PDZ domain specificity for NPT2A and PTHR is modulated by residues outside the binding pocket and by allosteric long-range communication. Despite significant progress, crystallization or cryo-electron microscopy to solve the structure of NHERF1 PDZ domains with the C-terminal -TRL motif of NPT2A will help distinguish binding determinants and improve our understanding of the origins of NHERF1 and other PDZ protein functional specificity. The recent finding that an internal PDZ region in NPT2A may regulate phosphate transport raises new questions about the structural determinant of this interaction and its impact on NHERF1. The influence of post-translational, site-specific phosphorylation on NHERF1 binding specificity and regulation of NPT2A-mediated phosphate uptake in health and disease remains uncharacterized. A combination of structural analysis, protein engineering and cell biology will be required to address these gaps in our understanding.

## Figures and Tables

**Table 1:** PDZ-selective NHERF1 interactions.

PDZ1	PDZ2
NPT2A (-ATRL^a^)	PKCα (-QSAV)
GRK6A (-PTRL)	GRK6A (-PTRL)
CFTR (-DTRL)	CFTR (-DTRL)
β2-AR (-DSSL)	TAZ/YAP65 (-LTWL)
PTHR (-ETVM)	PTHR (-ETVM)
PDGFR (-DSFL)	p38γ (-ETPL)
P2Y1 (-DTSL)	SGK1 (-DSFL)
PLCβ 1,2^b^,3	PLCβ 3 (-NTQL)

^a^-hydrophobic residues and Ser/Thr at position 0 and -2, respectively, are underline; ^b^-PLCβ 1 (-DTPL), PLCβ 2 (-ESPL).
